# Supplementary material for: Pyruvate Dehydrogenase Kinase 1 inhibition mediated oxidative phosphorylation enhancement in cartilage promotes osteoarthritis progression
Source: BMC Musculoskelet Disord. 2023 Jul 20;24:597. doi: 10.1186/s12891-023-06585-6 (PMC10357736; doi:10.1186/s12891-023-06585-6)
Supplement: Supplementary file 1 — Additional file 1. [file 12891_2023_6585_MOESM1_ESM.zip › MF.Gsea.1653627316242/GOMF_CYCLIC_NUCLEOTIDE_BINDING.html]

Details for gene set GOMF\_CYCLIC\_NUCLEOTIDE\_BINDING[GSEA]

|  || Dataset | 55457\_collapsed\_to\_symbols.cls.cls #OA\_versus\_normal.cls.cls #OA\_versus\_normal\_repos |
| Phenotype | cls.cls#OA\_versus\_normal\_repos |
| Upregulated in class | normal |
| GeneSet | GOMF\_CYCLIC\_NUCLEOTIDE\_BINDING |
| Enrichment Score (ES) | -0.4349718 |
| Normalized Enrichment Score (NES) | -1.4605794 |
| Nominal p-value | 0.03752039 |
| FDR q-value | 0.215678 |
| FWER p-Value | 1.0 |
Table: GSEA Results Summary

  

Fig 1: Enrichment plot: GOMF\_CYCLIC\_NUCLEOTIDE\_BINDING      
 Profile of the Running ES Score & Positions of GeneSet Members on the Rank Ordered List

  

| SYMBOL | TITLE | RANK IN GENE LIST | RANK METRIC SCORE | RUNNING ES | CORE ENRICHMENT || 1 | CNGA1 | cyclic nucleotide gated channel subunit alpha 1 [Source:HGNC Symbol;Acc:HGNC:2148] | 1988 | 0.362 | -0.1091 | No |
| 2 | POPDC3 | popeye domain containing 3 [Source:HGNC Symbol;Acc:HGNC:17649] | 2235 | 0.335 | -0.0863 | No |
| 3 | FKBP1B | FKBP prolyl isomerase 1B [Source:HGNC Symbol;Acc:HGNC:3712] | 2966 | 0.254 | -0.1112 | No |
| 4 | PDE11A | phosphodiesterase 11A [Source:HGNC Symbol;Acc:HGNC:8773] | 3090 | 0.241 | -0.0907 | No |
| 5 | CNP | "2',3'-cyclic nucleotide 3' phosphodiesterase [Source:HGNC Symbol;Acc:HGNC:2158]" | 4022 | 0.154 | -0.1437 | No |
| 6 | CNGB1 | cyclic nucleotide gated channel subunit beta 1 [Source:HGNC Symbol;Acc:HGNC:2151] | 4509 | 0.112 | -0.1674 | No |
| 7 | POPDC2 | popeye domain containing 2 [Source:HGNC Symbol;Acc:HGNC:17648] | 5578 | 0.026 | -0.2472 | No |
| 8 | HCN4 | hyperpolarization activated cyclic nucleotide gated potassium channel 4 [Source:HGNC Symbol;Acc:HGNC:16882] | 5590 | 0.025 | -0.2449 | No |
| 9 | PRKAR1B | protein kinase cAMP-dependent type I regulatory subunit beta [Source:HGNC Symbol;Acc:HGNC:9390] | 5806 | 0.008 | -0.2606 | No |
| 10 | CNGA3 | cyclic nucleotide gated channel subunit alpha 3 [Source:HGNC Symbol;Acc:HGNC:2150] | 6163 | -0.017 | -0.2861 | No |
| 11 | CNGB3 | cyclic nucleotide gated channel subunit beta 3 [Source:HGNC Symbol;Acc:HGNC:2153] | 6420 | -0.037 | -0.3014 | No |
| 12 | PDE6H | phosphodiesterase 6H [Source:HGNC Symbol;Acc:HGNC:8790] | 6877 | -0.069 | -0.3281 | No |
| 13 | PRKAR2B | protein kinase cAMP-dependent type II regulatory subunit beta [Source:HGNC Symbol;Acc:HGNC:9392] | 7034 | -0.082 | -0.3299 | No |
| 14 | HCN2 | hyperpolarization activated cyclic nucleotide gated potassium and sodium channel 2 [Source:HGNC Symbol;Acc:HGNC:4846] | 8387 | -0.185 | -0.4119 | Yes |
| 15 | PDE10A | phosphodiesterase 10A [Source:HGNC Symbol;Acc:HGNC:8772] | 8477 | -0.192 | -0.3948 | Yes |
| 16 | PDE4D | phosphodiesterase 4D [Source:HGNC Symbol;Acc:HGNC:8783] | 8797 | -0.216 | -0.3925 | Yes |
| 17 | KCNH1 | potassium voltage-gated channel subfamily H member 1 [Source:HGNC Symbol;Acc:HGNC:6250] | 8850 | -0.221 | -0.3688 | Yes |
| 18 | PDE2A | phosphodiesterase 2A [Source:HGNC Symbol;Acc:HGNC:8777] | 8872 | -0.223 | -0.3426 | Yes |
| 19 | PRKG2 | protein kinase cGMP-dependent 2 [Source:HGNC Symbol;Acc:HGNC:9416] | 9126 | -0.242 | -0.3320 | Yes |
| 20 | PDE6C | phosphodiesterase 6C [Source:HGNC Symbol;Acc:HGNC:8787] | 9633 | -0.286 | -0.3355 | Yes |
| 21 | SLC19A1 | solute carrier family 19 member 1 [Source:HGNC Symbol;Acc:HGNC:10937] | 9931 | -0.315 | -0.3192 | Yes |
| 22 | PDE4A | phosphodiesterase 4A [Source:HGNC Symbol;Acc:HGNC:8780] | 9957 | -0.317 | -0.2815 | Yes |
| 23 | PRKAR1A | protein kinase cAMP-dependent type I regulatory subunit alpha [Source:HGNC Symbol;Acc:HGNC:9388] | 10093 | -0.330 | -0.2506 | Yes |
| 24 | PDE5A | phosphodiesterase 5A [Source:HGNC Symbol;Acc:HGNC:8784] | 10441 | -0.366 | -0.2318 | Yes |
| 25 | PRKAR2A | protein kinase cAMP-dependent type II regulatory subunit alpha [Source:HGNC Symbol;Acc:HGNC:9391] | 10664 | -0.389 | -0.2003 | Yes |
| 26 | PRKG1 | protein kinase cGMP-dependent 1 [Source:HGNC Symbol;Acc:HGNC:9414] | 10776 | -0.403 | -0.1585 | Yes |
| 27 | RAPGEF2 | Rap guanine nucleotide exchange factor 2 [Source:HGNC Symbol;Acc:HGNC:16854] | 11041 | -0.435 | -0.1245 | Yes |
| 28 | PDE6G | phosphodiesterase 6G [Source:HGNC Symbol;Acc:HGNC:8789] | 11275 | -0.468 | -0.0840 | Yes |
| 29 | RAPGEF3 | Rap guanine nucleotide exchange factor 3 [Source:HGNC Symbol;Acc:HGNC:16629] | 11380 | -0.483 | -0.0316 | Yes |
| 30 | RAPGEF4 | Rap guanine nucleotide exchange factor 4 [Source:HGNC Symbol;Acc:HGNC:16626] | 11963 | -0.586 | -0.0035 | Yes |
| 31 | PDE4B | phosphodiesterase 4B [Source:HGNC Symbol;Acc:HGNC:8781] | 12057 | -0.609 | 0.0655 | Yes |
Table: GSEA details [plain text format]

  

Fig 2: GOMF\_CYCLIC\_NUCLEOTIDE\_BINDING      
 Blue-Pink O' Gram in the Space of the Analyzed GeneSet

  

Fig 3: GOMF\_CYCLIC\_NUCLEOTIDE\_BINDING: Random ES distribution      
 Gene set null distribution of ES for **GOMF\_CYCLIC\_NUCLEOTIDE\_BINDING**
